# The Structural and Physicochemical Properties of Isolated Starches from Canna (*Canna edulis* Ker.) Cultivated from Different Regions of China

**DOI:** 10.3390/gels12030267

**Published:** 2026-03-23

**Authors:** Junhong Feng, Qingling Luo, Peiling Liu, Cailin Niu, Yang Lu, Fayin Ye

**Affiliations:** 1 College of Food Science, Southwest University, Chongqing 400715, China; 18275689456@163.com (J.F.); luoqlng@163.com (Q.L.); 15283548452@163.com (P.L.); 13156355992@163.com (C.N.); ly1271761473@163.com (Y.L.); 2 Chongqing Engineering Research Center for Sweet Potato, Chongqing 400715, China; 3 Chongqing Key Laboratory of Speciality Food Co-Built by Sichuan and Chongqing, Chongqing 400715, China

**Keywords:** *Canna edulis* Ker., starch, structural properties, physicochemical properties, in vitro digestibility properties

## Abstract

Canna (*Canna edulis* Ker.) starch is an important non-conventional starch in global applications. In this study, the structural and physicochemical properties of canna starches extracted from four different geographical regions in China were investigated. The four starches (CES-DH, CES-MS, CES-YB, and CES-YX) exhibited relatively high total starch contents (82.51–93.22%). Apparent and true amylose contents varied markedly among samples, ranging from 31.44% to 43.62% and from 15.21% to 35.90%, respectively. Morphologically, the granules were oval and disc-shaped, with *D*_50_ values of 20.19–48.35 μm. CES-YX showed a distinct C-type pattern, while other starches exhibited B-type crystallinity, and relatively crystallinity values among samples were between 20.53% and 25.36%. IR absorbance ratios *R*_1047/1022_ and *R*_995/1022_ varied from 0.56 to 0.63 and from 1.15 to 1.26, respectively. Gelatinization temperatures and enthalpy revealed distinct thermal behaviors among the starches, corresponding to substantial differences in pasting properties with wide ranges in peak, breakdown, and setback viscosities. All starch pastes exhibited shear-thinning behaviors and weak gel characteristics. Notably, CES-YB demonstrated high potential as an effective food thickener and stabilizer, as distinguished by the high final viscosity and consistency coefficient (*K*), whereas the high amylose and resistant starch content in CES-YX made it a promising ingredient for low-glycemic-index food formulations. These findings provided a theoretical basis and practical guidance for the targeted utilization of canna starch in the food industry.

## 1. Introduction

Starch, a pivotal renewable biopolymer, finds extensive use in food, pharmaceutical, and industrial applications. Currently, maize, potato, cassava, and wheat dominate global starch production due to their high yields and established processing technologies [1]. However, the growing reliance on conventional sources, coupled with supply constraints, has spurred interest in alternative natural resources. Starches derived from “minor” plant sources, termed non-conventional starches, are consumed as staples in some rural developing regions but remain underutilized in industrial contexts [2]. These non-conventional sources are diverse, encompassing fruits and their by-products (e.g., seeds, pulp, and peels), rhizomatous and tuberous plants, cereals and pseudocereals, legumes, nuts, seaweeds, and food processing by-products [1]. Researchers have isolated and examined unconventional starches from various plant species and varieties, focusing on their structural and physicochemical properties. For instance, Yang et al. [3] found that starches from different banana cultivars contained 29.92–39.50% amylose and 44.74–55.43% resistant starch after cooking, exhibiting high peak viscosity (2248–2897 cP) with low breakdown (556–960 cP) and setback values (583–864 cP), suggesting their potential as alternatives to chemically cross-linked starches in food systems requiring viscosity stability. Zhang et al. [4] reported that starches from five jackfruit cultivars demonstrated high purity (>99%) and amylose contents of 26.41–38.24%, with significant cultivar-dependent differences in swelling, pasting, thermal, and crystalline properties, primarily influenced by amylose content and particle size. Yan et al. [5] investigated 30 sorghum starches with apparent amylose contents of 7.42–36.44%, revealing that a higher amylose content reduced swelling, peak viscosity, and gelatinization enthalpy while increasing setback, underscoring amylose as a critical determinant of functionality.

Among non-conventional starch sources, *Canna edulis* Ker. (family Cannaceae, genus Canna) stands out as an herbaceous plant native to South America’s Andean region [6]. It thrives in tropical and subtropical zones, including South America and Southeast Asia [7]. Prior research highlights its nitrogen use efficiency, drought tolerance, and productivity, enabling cultivation under forest canopies or low-light conditions [8]. The crop boasts a global average yield of 30–40 t/ha, with peak yields of 85 t/ha in Colombia and 45 t/ha in China [9]. Its starch yield typically ranges from 4.1 to 6.2 t/ha, influenced by environmental and agronomic factors [9]. The rhizome contains 70–80% starch (dry basis) [10]. Commercially, canna starch is supplied by manufacturers, such as Kono Chem Co., Ltd. (Xi’an, China), Guizhou Xinchang Starch Technology Co., Ltd. (Southwest Guizhou Autonomous Prefecture, China), and Guangxi Fuwang Technology Co., Ltd. (Nanning, China). It serves as a food ingredient in starch noodles and wheat noodles and as a thickener in traditional Chinese dishes.

Several studies have explored the composition, physicochemical properties, and applications of canna starch. Thitipraphunkul et al. [6] found that canna starch granules were round-to-oval, with smooth surfaces and particle sizes of 10–100 μm. Yaruro Cáceres et al. [11] reported total starch contents of 73.50–85.56% and paste clarity values of 43.09–58.89% for four Colombian canna starches, with peak viscosity values of 13,480–15,606 cP and setback values of 3358–4960 cP, supporting their use as thickening agents in baked products. Hung and Morita [12] showed that canna starch gel was unstable during frozen storage, with net syneresis increasing from 72.6% to 77.0% over 24–96 h. Huang et al. [13] found that the resistant starch contents of native, gelatinized, and retrograded canna starch were 93.8%, 9.4%, and 12.7%, respectively. Umam et al. [14] demonstrated that yogurt drinks prepared with 0.1% (*w*/*w*) canna starch as a hydrocolloid substitute exhibited viscosity (80.1 cP) and sensory properties comparable to those of a control containing 0.2% (*w*/*w*) carboxymethyl cellulose. Cao et al. [15] added 24% (*w*/*w*) canna starch to wheat flour, improving flour characteristics, rheological properties, and noodle texture while reducing the expected glycemic index of semi-dried noodles from 41.16 to 39.90. These findings highlight the functional potential of canna starch in both conventional and health-oriented food systems. However, the currently available studies mainly focused on certain individual aspects, and information covering the structural characteristics, physicochemical properties, and digestion behavior of canna starch remains limited. Therefore, further investigation is needed to better understand the techno-functional properties of canna starch.

Thus, to meet the high demand of new-source starches and extend the utilization of canna starch, this study aims to examine the chemical composition, molecular structure and physicochemical properties and in vitro digestion characteristics of isolated starches from canna produced in four major production areas in China. The results from the present study will be useful for guiding potential value-added utilization of canna starch in both food and non-food industries.

## 2. Results and Discussion

### 2.1. Chemical Composition

As shown in Table 1, the chemical composition of canna starches from four different geographical origins showed significant differences (*p* < 0.05). The moisture content in the starch samples was mainly related to non-starch components, which affected the water-binding capacity of the extracted starch product under equilibrium conditions. A higher moisture content was determined in CES-MS. This might be associated with its higher ash content, as mineral components were hydrophilic and might enhance the water-binding capacity of starch granules under the same storage conditions. The moisture content of the canna starches varied from 11.87% to 18.19%, which was consistent with previously reported results of 18.17% [16] and 13.58–14.22% [11], respectively. The starch content was an indicator of the starch purity. The starch content of the four samples varied, which was mainly related to the cultivar and extraction process [17]. The maximal starch content was determined in CES-YX (93.22%), indicating that impurities were most effectively removed during its extraction process.

Amylose content, which showed an important influence on the physicochemical properties (such as gelatinization, retrogradation, and rheological) of starch, has been quantified by various methods, such as the iodine colorimetric method, amperometric titration, differential scanning calorimetry, the concanavalin A method, gel permeation chromatography, and high-performance anion-exchange chromatography. Due to the differences in measurement principles, amylose values obtained using different methods for the same sample might vary [18]. In our study, the four canna starches showed greater apparent amylose content (AAC) than the true amylose content (TAC) values. AAC was determined by the iodine colorimetric method, which is based on the principle that linear segments in starch (amylose and the long side chains of amylopectin) formed a blue complex with iodine. TAC was determined using the GPC method, which clearly distinguished the long chains of amylose (referring to the portion with a degree of polymerization > 100) from the short chains of amylopectin on the elution curve. The TAC value was obtained by calculating the ratio of the area of DP > 100 to the peak area [19]. Thitipraphunkul et al. [6] determined the iodine affinity of three canna starches by using amperometric titration and calculated that AAC values (21–28%) were higher than their TAC values (19–25%). In our study, the AAC values of the four canna starches ranged from 31.44% to 43.62%, which was comparable to a previously reported result of 38.64% [20].

Notably, lipids, proteins, and ash constituted minor components in canna starch (<1%), and their contents showed significant differences (*p* < 0.05), which were probably related to the variety and cultivation conditions. Consistent with a previous study, Andrade-Mahecha, Tapia-Blacido and Menegalli [21] found that two canna starch samples from Brazil and Colombia had crude protein, lipid, and ash contents ranging from 0.60% to 0.81%, 0.05% to 0.15%, and 0.22% to 0.53%, respectively.

### 2.2. Color Analysis

As displayed in Table 1, the color attributes of the four canna starches showed significant differences (*p* < 0.05). The L* value reflected the lightness of the sample. A greater L* value indicated that starch granule showed a smoother surface and a higher degree of surface impurity removal. In our study, the L* values of the canna starches (92.63–97.79) were greater than previous results (90.50–91.99) reported by Yaruro Cáceres et al. [11]. This difference might be attributed to variations in geographical origins and extraction methods, suggesting that the canna starches in this study possessed high surface smoothness and cleanliness. a* (+, redness; −, greenness) reflected the red–green balance of the sample. The a* values of all samples were close to 0, suggesting that the color was near neutral. Specifically, the a* values of CES-YB and CES-DH were positive, showing a red hue, which was probably related to browning reactions that occurred during the extraction process [22]. In contrast, the a* values of CES-MS and CES-YX were negative, showing a green hue, which might be associated with the residual natural pigments that were not completely removed during the extraction process. b* (+, yellowness; −, blueness) reflected the yellow–blue balance of the sample. The b* values of all samples were positive, indicating a yellowish hue, which might be related to the residual natural pigments (e.g., carotenoids) in the extracted starch [23]. The whiteness index (*WI*), which was calculated based on the L*, a*, and b* values, varied from 0 to 100. A value of 100 represented pure white, while a value of 0 represented blackness. The *WI* values of all samples were above 90, probably suggesting that all samples had high purity and that impurities were effectively removed during the starch extraction process.

### 2.3. Morphological Analysis

**Figure 1:**
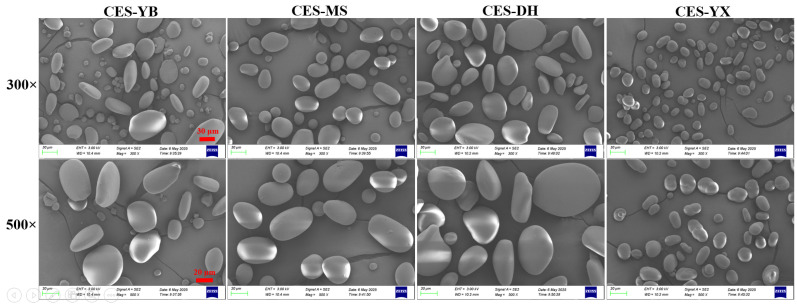
The morphologies of canna starch granules from four different geographical regions under scanning electron microscope (SEM) with 300× and 500× magnification.

Scanning electron microscope images of the four canna starches are shown in Figure 1. At a magnification of 300×, all samples consisted of starch granules of various sizes. After processing with the ImageJ software (version 1.54, National Institutes of Health, Bethesda, MD, USA), the overall size range of the canna starch granules was as follows: 4.29–79.35 μm (CES-YB), 5.28–60.37 μm (CES-MS), 15.42–84.75 μm (CES-DH), and 4.40–33.63 μm (CES-YX). The particle size of the CES-DH starch was generally greater than the others. Based on the length of the long axis of the particles, starch granules were empirically classified into small granules (<10 μm), medium granules (10–30 μm), and large granules (>30 μm) [24]. CES-YX mainly consisted of small and medium granules, while the other three samples comprised small, medium, and large granules. As examined more closely (500× magnification), the large granules were oval or disc-shaped with smooth surfaces; the medium granules were round, oval, or flattened, with some having a dimpled surface; and the small granules were spherical or slightly angular, with smooth surfaces. This might be related to the variety, its geographic origin, and the presence of small granules that were not recovered during the starch extraction process. Yaruro Cáceres et al. [11] noted that starch granules from four types of canna starch in Colombia were disc- or oval-shaped with smooth surfaces, and some granules were round or irregular in shape. Castillo-Paz et al. [25] reported that Colombian canna starch contained spherical, ellipsoidal, and ovoid granules, with a smooth surface. The differences in the size and shape distribution of the granules might influence the functional properties of the starch.

### 2.4. Particle Size Distribution

**Figure 2:**
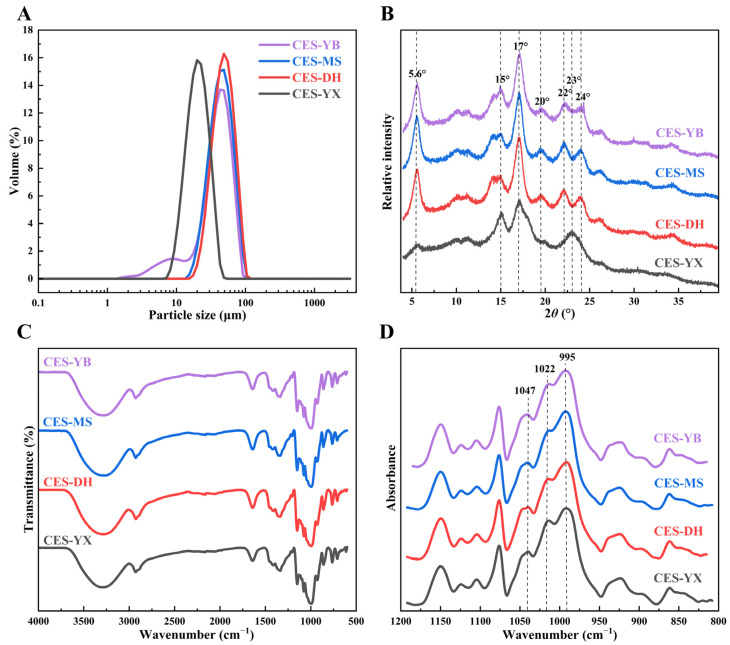
Particle size distribution (**A**), X-ray diffraction patterns (**B**), FT-IR spectra (**C**) and deconvoluted FT-IR spectra (**D**) of canna starches from four different geographical regions.

Previous studies indicated that particle size affected the physicochemical properties of tuber starches. As shown in Figure 2A, the particle size distribution results were consistent with the SEM observations. Among them, the particle size distribution curve of CES-YX was positioned at the leftmost and exhibited a unimodal distribution with a peak at 19.90 μm. CES-MS and CES-DH also displayed unimodal distributions, with peaks at 47.28 μm and 48.62 μm, respectively. However, CES-YB showed a bimodal distribution, with the main peak at 45.05 μm and a small shoulder peak at 9.24 μm. The particle size distribution of CES-YB was similar to the typical A-type and B-type particle bimodal distribution, which was found in wheat starch [26]. The bimodal distribution in CES-YB might reflect the inherent size heterogeneity of the native starch granules and potential physical particle agglomeration. Andrade-Mahecha, Tapia-Blacido and Menegalli [21] reported that canna starch from Brazil exhibited a unimodal distribution, with granule sizes in the range of 7.5–90.5 μm, while Colombian commercial canna starch showed a bimodal distribution pattern, i.e., one with a particle size ranging from 19 μm to 121 μm and another with sizes > 200 μm (probably agglomerated starch granules). Furthermore, the particle size distribution parameters of the four canna starches (Table 1) showed significant differences (*p* < 0.05). *D*_10_, *D*_50_, and *D*_90_ represented the particle diameters at cumulative volume percentages of 10%, 50%, and 90%, respectively [27]. The value of *D*_50_ (median particle size) of CES-YX was approximately a half of the other three samples. *Span* measured the dispersion of the particle size distribution [27]. Among the four samples, CES-YB showed the maximal *Span* value (1.39), suggesting a more dispersed particle size distribution and greater variation in granule size. *D*_[4,3]_, the volume-weighted average particle size (De Brouckere mean diameter), was sensitive to large particles and was commonly used as the mean particle size in laser-diffraction-based volume distribution. *D*_[3,2]_, the surface-area-weighted average particle size (Sauter mean diameter), was related to the specific surface area [28]. The *D*_[4,3]_ and *D*_[3,2]_ values of the canna starches varied from 21.16 μm (CES-YX) to 50.42 μm (CES-DH) and from 18.81 μm (CES-YX) to 44.83 μm (CES-DH), respectively. These values showed trends consistent with the *D*_50_ value. All the results are reported as equivalent spherical diameters, as the laser diffraction method assumed spherical geometry via the Mie theory. Considering the ellipsoidal morphology of the granules (Figure 1), these values were intended for comparative analysis.

### 2.5. Structural Characteristics

#### 2.5.1. XRD Analysis

Figure 2B shows the XRD patterns of the four canna starches. Based on the positions of the diffraction bands, starch was classified into A-type, B-type, and C-type [29]. Specifically, A-type starch exhibited two strong diffraction bands at approximately 15° and 23° (2*θ*), with double diffraction bands near 17° and 18° (2*θ*). B-type starch had a strong diffraction band at around 17° (2*θ*), small diffraction bands at 15°, 22°, and 24° (2*θ*), and a characteristic band at 5.6° (2*θ*). C-type starch displayed crystallization features of both A-type and B-type starch. Based on the ratio of A-type to B-type polymorphs, C-type starch was further divided into C_A_-type (closer to A-type), C_B_-type (closer to B-type), and C_C_-type (typical C-type) [29]. In this study, CES-YB, CES-MS, and CES-DH were classified as B-type starch, which displayed characteristic diffraction bands at 5.6° and 15° (2*θ*), a single diffraction band at 17° (2*θ*), and double diffraction bands between 22° and 24° (2*θ*). Previous studies identified canna starch as B-type [11,13]. In contrast, CES-YX was classified as C_C_-type, showing a weak diffraction band at 5.6° (2*θ*) and single diffraction bands at 15°, 17°, and 23° (2*θ*). Similarly, XRD patterns indicated C_A_-type canna starch from Indonesia [20]. The difference in crystalline pattern of CES-YX might be related to its higher apparent amylose content and lower lipid content, which could influence the packing of amylopectin double helices. Notably, CES-YB, CES-MS, and CES-DH exhibited diffraction bands at approximately 20° (2*θ*), indicating the potential presence of a V-type crystalline structure, which was associated with starch–lipid complexes [30]. However, the diffraction band at 20° (2*θ*) in CES-YX was weak and only faintly observed, probably due to its low lipid content (0.22%).

As presented in Table 2, the RC values of the four canna starches showed significant differences (*p* < 0.05). The RC value of CES-MS was greater than that of the other samples, which might be related to its lower AAC value and higher lipid content. It was reported that amylopectin was the determining factor for starch crystallinity, while amylose disrupted the crystal packing structure of amylopectin [31]. A decrease in RC was also found after the removal of starch-granule-associated lipids from normal wheat starch [30]. CES-YX showed the lowest RC value, which was probably due to its high AAC and small granule size. Zhang et al. [4] found a significant positive correlation between particle size and RC of starches from five jackfruit cultivars (*r* = 0.87, *p* < 0.01). Xu et al. [32] observed that the particle size and the RC of cassava starch gradually increased as the growth period of cassava starch increased. In this study, the RC values of the four canna starches ranged from 20.53% to 25.36%, which was comparable to previously reported RC values of 25.70% [13] and 22.18% [33].

#### 2.5.2. Short-Range Ordered Structure of Starches

The FTIR spectra of the four canna starches are presented in Figure 2C. The positions of characteristic bands in the wavenumber range 4000–600 cm^−1^ were similar among all samples, indicating that they shared similar functional groups and chemical bonds. Specifically, the broad band at 3700–3000 cm^−1^ was attributed to the stretching vibration of O-H bonds; the band at 2935 cm^−1^ was attributed to the stretching vibration of -CH_2_- bonds; the band at 1645 cm^−1^ was probably from the bending vibration of O-H bonds in tightly bound water molecules in the starch [34]. The region in the 1200–800 cm^−1^ range corresponded to the fingerprint region of starch, which was mainly associated with the stretching vibrations of C-C, C-O, and C-O-H bonds, as well as the bending vibration of C-O-H bonds [34]. In this region, the characteristic absorption bands at 1047, 1022, and 995 cm^−1^, which were sensitive to the conformational changes of starch, were associated with the crystalline region, the amorphous region, and the double-helix structure of starch, respectively [35]. To further investigate the short-range ordered structures of the four canna starches, the 1200–800 cm^−1^ region of the original spectrum was deconvoluted and is depicted in Figure 2D. Further, the infrared absorbance ratios *R*_1047/1022_ and *R*_995/1022_, calculated from the deconvoluted spectra, were used to evaluate the degree of short-range order (DO) and the degree of double helices (DD) of the starches, respectively [35]. As presented in Table 2, the four starches showed significant differences in the DO and DD values (*p* < 0.05). Evidently, the trends in short-range order and RC were not completely consistent and were probably attributed to the asynchronous formation of short-range and long-range ordered structures [31]. CES-MS showed a higher DO than the other samples, indicating that its external regions were more ordered, which might be associated with its lower AAC. Yan et al. [5] found that the DO of sorghum starch was negatively correlated with AAC (*r* = −0.70, *p* ≤ 0.001), suggesting that the order of sorghum starch was influenced by AAC. Meanwhile, the DD of CES-MS was also significantly higher than that of the other samples (*p* < 0.05), indicating a stable double-helix structure, which was probably related to its lower AAC and longer growth period. Li et al. [36] pointed out that the double-helix structures were formed either by a pair of amylopectin side-chain clusters or by the interaction between inter-block amylopectin chains and amylose clusters. Shi et al. [37] found that the double-helix order of millet starch decreased as AAC increased. Xu et al. [32] reported that the *R*_995/1022_ ratio of cassava starch increased as the growth period progressed, reflecting an enhanced structural order of starch and improved hydration of the double-helix structure.

#### 2.5.3. Chain Length Distribution of Starches

**Figure 3:**
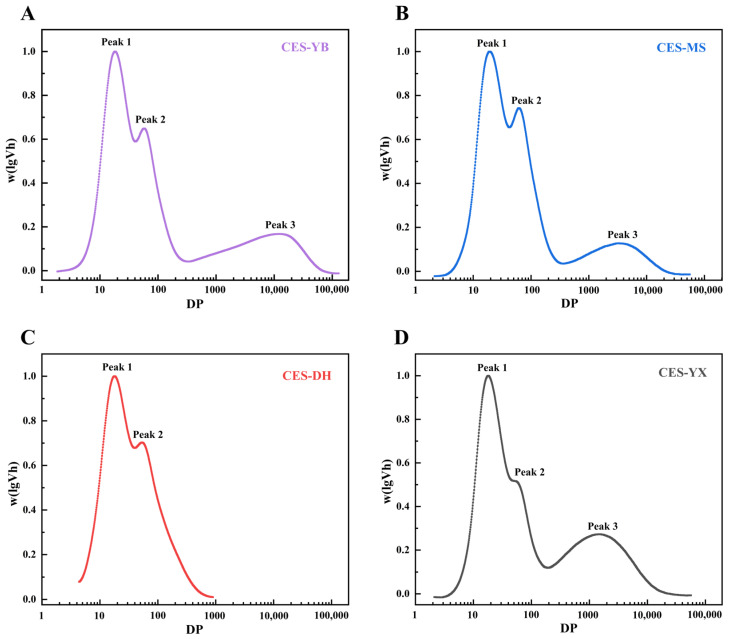
Chain length distribution of debranched canna starches from four different geographical regions in China: (**A**) CES-YB; (**B**) CES-MS; (**C**) CES-DH; (**D**) CES-YX. *w* (lg*V*_h_), the logarithmic distribution of hydrodynamic volume; DP, the degree of polymerization.

The chain length distribution curves of the four debranched canna starches are presented in Figure 3A–D. CES-YB, CES-MS, and CES-YX exhibited trimodal distribution patterns, whereas CES-DH displayed a bimodal distribution. Peak 1 corresponded to amylopectin short chains with a degree of polymerization (DP) ≤ 30, which were mainly located within the amorphous and crystalline lamellae of native starch granules. Peak 2 represented amylopectin long chains with DP values ranging from 30 to 100, which extended across multiple lamellar structures [19]. The branching degree of amylopectin was evaluated using the area ratio of Peak 1 to Peak 2, with a higher ratio indicating a greater degree of branching [13]. As shown in Table 2, CES-DH exhibited the highest proportion of amylopectin short chains and the greatest branching degree, which might be associated with its low amylose content. Similarly, Gong et al. [38] reported that debranched glutinous rice starch with a low amylose content also presented a bimodal chain length distribution. The chains with DP < 100 were classified as amylopectin, whereas those with DP ≥ 100 were regarded as amylose [19]. As discussed in Section 2.1, the TAC values determined by GPC for all samples were lower than the AAC values obtained using the iodine colorimetric method. This discrepancy might be attributed to the tendency of long amylopectin chains to bind iodine, leading to an overestimation of the amylose content [18]. From a functional perspective, AAC was often considered more relevant because iodine-binding linear segments, including both amylose and long amylopectin chains, could influence important physicochemical properties such as gelatinization, retrogradation, and rheological behaviors.

### 2.6. Techno-Functional Properties

#### 2.6.1. SOL and SP

**Figure 4:**
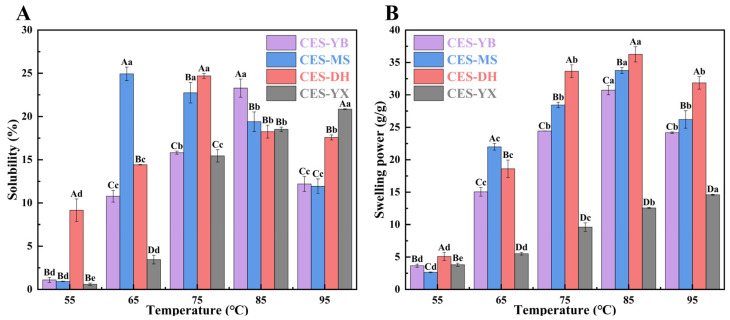
Solubility (**A**) and swelling power (**B**) of canna starches from four different geographical regions at 55 °C, 65 °C, 75 °C, 85 °C, and 95 °C, respectively. Different uppercase letters indicate significant differences among starch samples at the same temperature, while different lowercase letters indicate significant differences among temperatures within the same starch (*p* < 0.05).

Figure 4A shows the SOL of four canna starches. The SOL of CES-YX increased as the temperature increased from 55 °C to 95 °C, and CES-YX exhibited a significantly greater SOL value than the other samples at 95 °C (*p* < 0.05). This might be associated with its high amylose content and low DD. When starch was heated with sufficient water, the hydrogen bonds that stabilized the double helices were disrupted and replaced by starch–water hydrogen bonds, resulting in granule swelling and leaching of soluble starch (mainly amylose) [39]. In contrast, CES-YB, CES-MS, and CES-DH exhibited an initial increase in SOL followed by a decrease with further temperature rise, with peak values observed at 65 °C for CES-MS, 75 °C for CES-DH, and 85 °C for CES-YB, respectively. The reduction in SOL at elevated temperatures was probably associated with amylose–lipid complex formation and denaturation of granule-associated proteins, which hindered the solubilization. Ashogbon [40] measured the water solubility index of cassava starch over the temperature range of 55–95 °C and found that it showed an increase up to 65 °C, then a decrease at 75 °C, followed by a sharp increase at higher temperatures, and they concluded that the decrease at 75 °C was associated with the effects of residual proteins, lipids, and temperature-induced complexes.

Figure 4B shows the SP of the four canna starches. With the increase in temperature from 55 °C to 95 °C, the SP of CES-YX continuously increased, while the SP of CES-YB, CES-MS and CES-DH increased in the temperature range of 55–85 °C and then decreased at 95 °C. Notably, CES-YX showed significantly lower SP than the other samples between 65 °C and 95 °C (*p* < 0.05), which was probably due to its high amylose content and small granule size. At 75–95 °C, CES-DH showed a significantly greater SP value than the other samples (*p* < 0.05), which might be attributed to its large granule size. A previous study showed that amylose restricted starch granule swelling and maintained the integrity of swollen granules [41]. Zhang et al. [4] indicated that larger starch granules swelled more rapidly than smaller counterparts during heating in water, resulting in higher SP. For CES-YB, CES-MS and CES-DH, the decline in SP at 95 °C was probably due to the loss of granule integrity after extensive swelling [42].

#### 2.6.2. Thermal Properties of Starches

**Figure 5:**
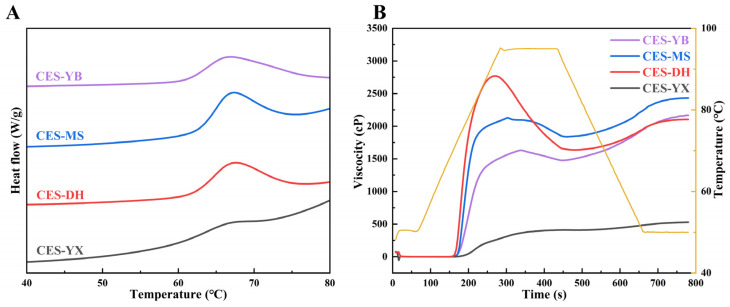
DSC thermograms (**A**) and RVA curves (**B**) of canna starches from four different geographical regions in China.

As shown in Figure 5A, the four canna starches exhibited smooth endothermic curves with distinct peak positions and widths. The thermal parameters, including the onset temperature (*T*_o_), peak temperature (*T*_p_), conclusion temperature (*T*_c_), gelatinization temperature range (Δ*T* = *T*_c_ − *T*_o_), and enthalpy of gelatinization (Δ*H*_g_), are presented in Table 3. In this study, the gelatinization temperatures were *T*_o_ (61.30–63.13 °C), *T*_p_ (66.68–67.61 °C), *T*_c_ (70.96–77.36 °C), and Δ*T* (9.14–16.06 °C), which were comparable to those reported for native canna starch [13]. Among them, CES-MS had the highest *T*_o_, which was probably due to its highest lipid content (0.74%). Lipids conferred hydrophobicity of the starch granule surface, thereby restricting water absorption and swelling during heating and increasing the gelatinization temperature [43]. No significant differences in *T*_p_ were observed among the samples. Variations in *T*_c_ values were observed among the samples, and CES-YB showed a significantly greater *T*_c_ value than the other samples (*p* < 0.05). This was probably due to its dispersed particle size distribution and relatively low RC, as *T*_c_ mainly reflected the subcrystalline regions of starch [44]. Accordingly, CES-YB exhibited the widest Δ*T*, which might be related to its uneven particle size distribution with the maximal *Span* value (1.39). Δ*T* reflected the stability and heterogeneity of crystalline regions in starch granules, and a wider Δ*T* indicated the presence of crystallites with different thermal stabilities [5]. Jang et al. [45] reported that the variations in Δ*T* were linked to the differences in granule size and uniformity within indica rice starches.

Overall, the Δ*H*_g_ of the four canna starches was relatively low, with values falling between 0.43 J/g and 3.53 J/g. Among them, CES-YB exhibited a significantly higher Δ*H*_g_ than other samples (*p* < 0.05), indicating that more energy was needed to break the intermolecular bonds in the starch granules, which was probably due to its uneven particle size distribution and lower proportion of short branch chains in amylopectin. Δ*H*_g_ has been regarded as an indicator of the quantity and quality of starch crystallites, since the endothermic transition corresponded to the disruption of microcrystalline regions [46]. Zhu et al. [41] reported that Δ*H*_g_ reflected the loss of double-helical structures during starch gelatinization, and a high proportion of short branch chains in amylopectin could reduce the number of double helices. In contrast, CES-YX showed a significantly lower Δ*H*_g_ value than the other samples (*p* < 0.05), which might be associated with its small granule size, high amylose content, and low RC, DO, and DD. This result suggested that fewer or less ordered double helices were present in the crystalline regions of CES-YX, indicating a relatively low level of starch granule organization and weaker crystalline stability [47]. Kong et al. [39] reported that, among five loquat starches, Δ*H*_g_ was correlated with granule size, RC and amylose content; the sample with higher Δ*H*_g_ had greater RC and larger granules, while the lowest Δ*H*_g_ was associated with amylose-rich and poorly crystalline starch. Shi et al. [37] found that the Δ*H*_g_ of millet starch was negatively correlated with AAC, indicating that starches with higher AAC had a less ordered molecular structure, i.e., fewer crystallites and double helices. Although CES-MS exhibited the highest RC, DO, and DD, it showed a relatively low Δ*H*_g_ value. This might be explained by the water-limited testing conditions during DSC heating, under which Δ*H*_g_ did not fully reflect the melting of crystalline regions or dissociation of double helices, and stable crystallites were less disrupted [46].

#### 2.6.3. Pasting Properties of Starches

The pasting curves of the four canna starches are depicted in Figure 5B. The four canna starches exhibited three distinct pasting patterns characterized by differences in viscosity development, peak formation, and starch paste stability during heating and shear and viscosity recovery during cooling. Pasting parameters, including peak (PV), trough (TV), breakdown (BD = PV − TV), final (FV), and setback (SB = FV − TV) viscosities, are summarized in Table 3. PV reflected the water absorption capacity and swelling degree of starch granules [37]. CES-DH exhibited a rapid increase in viscosity and showed a significantly higher PV (2721.50 cP) than the other samples (*p* < 0.05), which might be related to its lower amylose content and larger granule size. This was consistent with its highest SP at 75–95 °C. In contrast, CES-YX showed a slow viscosity development without an obvious peak and exhibited the lowest PV value (399.50 cP), which was probably due to its high amylose content and small granule size. During heating, the pasting behaviors of starch involved water absorption, granule swelling, disruption of ordered structures, and the formation of starch paste with increased viscosity, with pasting properties determined by the extent of swelling, the proportion of swollen granules, and the interaction among starch chains [48]. TV reflected the shear resistance of swollen starch granules at high temperature. CES-MS showed a significantly higher TV (1847.00 cP) than the other samples (*p* < 0.05), indicating stronger shear resistance during the holding stage. BD measured the degree of breakdown of swollen starch under shear and heat, reflecting the thermal stability of the starch paste. CES-DH showed a pronounced decrease in viscosity, displaying the highest BD (1088.50 cP), probably due to its large granule size, which was consistent with its highest SP. In contrast, CES-YX showed the lowest BD (23.50 cP), indicating good thermal stability, as its small granule size limited the swelling progress. During the holding stage at 95 °C, the paste viscosity began to decrease due to granule disintegration, leaching of starch chains (mainly amylose) into the medium, and molecular alignment [48]. FV reflected the thickening ability of the starch paste after cooling. CES-MS showed the highest FV (2413.50 cP), suggesting strong thickening ability. SB indicated the extent to which the viscosity of the starch paste increased during cooling, reflecting the short-term retrogradation characteristics. CES-YB exhibited a pronounced viscosity recovery upon cooling, showing a significantly higher SB (714.00 cP) than the other samples (*p* < 0.05), indicating stronger molecular interactions (e.g., rearrangement and recrystallization) between amylose and amylopectin [37]. Starches with high amylose content generally exhibited rapid short-term retrogradation tendency, resulting in high SB values [46]. However, CES-YX had the lowest SB (142.50 cP), regardless of its highest amylose content. It was probably attributed to its smallest granule size that limited the granule swelling (Figure 4B). Compared with hard jackfruit seed starch, a lower SB value was observed in soft jackfruit seed starch due to its smaller particle diameter [49]. Overall, the relatively low pasting viscosities of CES-YX suggested that, in addition to its small granule size, a higher degree of disintegration of swollen starch granules might also contribute to its limited viscosity development during pasting [47].

#### 2.6.4. Steady Flow Properties of Starch Pastes

**Figure 6:**
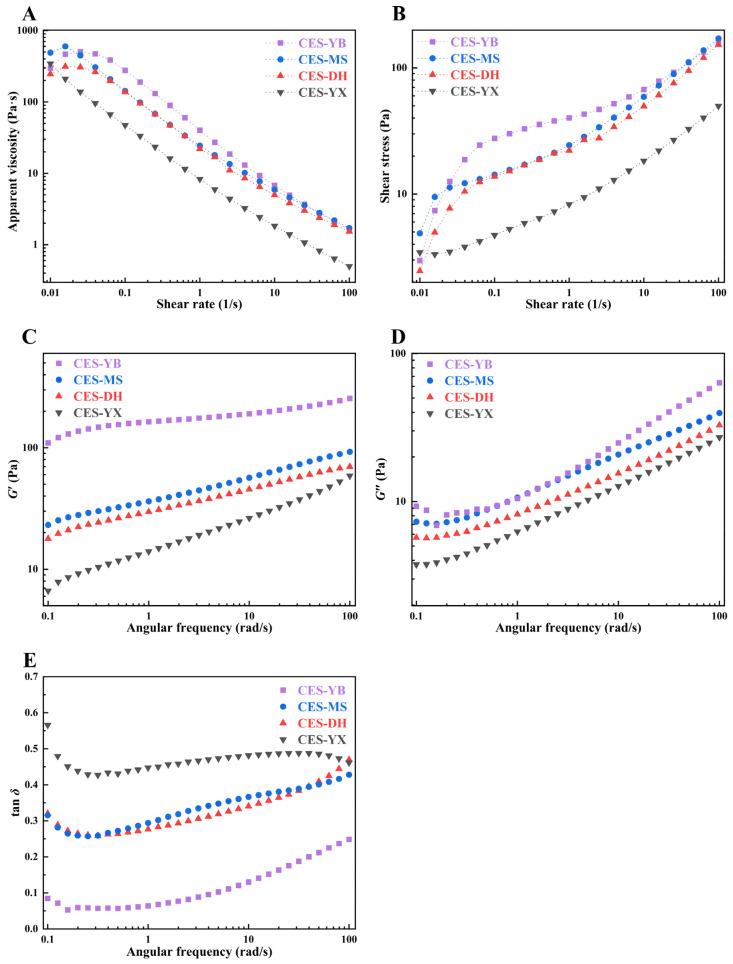
Rheological properties of canna starch pastes from four different geographical regions in China. Apparent viscosity as a function of shear rate (**A**); stress as a function of shear rate (**B**); storage modulus (*G*′) as a function of angular frequency (**C**); loss modulus (*G*″) as a function of angular frequency (**D**); tan *δ* (*G*″/*G*′) as a function of angular frequency (**E**).

As shown in Figure 6A, the apparent viscosity of the canna starch pastes showed a decreasing trend overall as the shear rate increased. Thus, they exhibited typical shear-thinning behaviors and were classified as pseudoplastic fluids. Specially, CES-YB, CES-MS, and CES-DH showed a slight viscosity increase at low shear rates (0.01–0.1 s^−1^). This might be related to the delayed structural reconstruction under weak shear conditions [50]. Overall, the shear stress of all samples increased with the increase in shear rate (Figure 6B). CES-YB, CES-MS, and CES-DH exhibited a pronounced increase in shear stress at low shear rates (0.01–0.1 s^−1^), indicating high resistance to flow initiation, which was consistent with their increased apparent viscosity in this region. As shown in Table 3, the coefficient of determination (*R*^2^) ranged from 0.984 to 1.000, indicating that the Herschel–Bulkley model fitted the steady flow behaviors. The flow behavior index (*n*) described the difference between non-Newtonian fluid and Newtonian fluid. When *n* equaled 1, the system exhibited Newtonian behavior, whereas *n* > 1 and 0 < *n* < 1 corresponded to dilatant and pseudoplastic behaviors, respectively [51]. As *n* approached 0, the shear-thinning effect became more pronounced, indicating a highly shear-sensitive internal structure. All starch pastes exhibited *n* values below 1 (0.38–0.52), confirming their pseudoplastic behaviors. Notably, CES-YB showed a significantly lower *n* value than the other samples (*p* < 0.05), reflecting its stronger shear-thinning capacity. The yield stress (*τ*_0_) represented the stress required to initiate flow; the consistency coefficient (*K*) reflected the viscosity level of the starch paste and was associated with the consistency of the system [3]. CES-YB exhibited significantly greater *τ*_0_ and *K* values than the other samples (*p* < 0.05), indicating a stronger resistance to flow initiation and a more developed internal paste structure. In contrast, CES-YX showed the lowest *τ*_0_ and *K* values, suggesting a weaker internal structure and reduced viscosity. This behavior might be partially attributed to its small granule size that limited granule swelling and intergranular interactions. Abelti et al. [52] reported that differences in the consistency index between maize and potato were attributed to variations in granule size and shape, amylose content, and lipid content.

#### 2.6.5. Dynamic Viscoelastic Properties of Starch Pastes

Frequency sweep measurements of the four canna starch pastes were performed within the LVR at a constant strain of 2%, and the corresponding moduli were recorded. The storage modulus (*G*′) and loss modulus (*G*″) represented the elasticity and viscosity of the system, respectively [44]. As shown in Figure 6C,D, both *G*′ and *G*″ of all samples overall increased with increasing angular frequency, indicating frequency-dependent behavior. Specifically, the G″ values of CES-YB, CES-MS, and CES-DH decreased slightly in the low-frequency region (0.1–1 rad/s). In addition, *G*′ consistently exceeded *G*″ for all canna starch pastes, indicating the solid-like characteristics of weak gels [44]. CES-YB had greater *G*′ values than the other samples over the entire frequency range, which was consistent with its highest SB value. To further evaluate the angular frequency dependence of the modulus, the power-law model was employed to fit the data, and the corresponding parameters are summarized in Table 3. The *R*^2^ exceeded 0.975, indicating good fit effect for all samples. CES-YB showed a higher *K*′ value than the other samples, which corresponded to its higher *G*′ values. The exponent *a* reflected the extent to which the flow behavior deviated from Newtonian behavior, with values closer to 0 indicating more elastic characteristics and values approaching 1 indicating more viscous behaviors [53]. CES-YB showed the lowest *a* value, confirming its enhanced elasticity. Tan *δ* was used to characterize the damping properties of the materials, and a lower tan *δ* indicated the formation of a more developed gel network, while a higher tan *δ* reflected a more viscous and loose structured system [53]. As shown in Figure 6E, all canna starch pastes showed tan *δ* values below 1 over the entire frequency range, indicating the dominance of elastic behavior [34]. CES-YB exhibited markedly lower tan *δ* values than the other samples, indicating a stronger gel network, which was consistent with its highest *K* value (24.42 Pa·s^n^), *K*′ value (94.08 Pa), and SB (714.00 cP).

#### 2.6.6. Texture Properties of Starch Gels

The textural properties of the four canna starch gels are summarized in Table 3. Except for springiness, all other parameters exhibited significant differences between samples (*p* < 0.05), reflecting pronounced differences in their gelation behaviors. Hardness was defined as the maximum force required to deform the gel during the first compression, reflecting the overall strength and rigidity of the gel network. Compared to the other samples, CES-DH showed higher hardness (3250.83 g), indicating a strong interconnected gel structure. This was mainly related to its rapid granule swelling during the gelatinization heating process, which formed the basis of its gel structure [54]. Springiness described the ability of a gel to recover its original height after the first compression, reflecting the quantity of the helical structure. CES-DH exhibited higher springiness than the other samples, suggesting a more elastic and ordered three-dimensional network that favored reversible deformation rather than irreversible structural breakdown [55]. This was probably due to its higher proportion of long chains in amylopectin, as the internal long chains of amylopectin participated in intermolecular interactions during retrogradation, resulting in a compact starch gel microstructure [56]. Adhesiveness referred to the work required to overcome the attractive forces between the gel surface and the probe, reflecting the stickiness of the gel. CES-DH showed higher adhesiveness than the other samples, which was consistent with its highest PV value and greatest SP at 75–95 °C that resulted in increased paste viscosity. Cohesiveness reflected the internal structural integrity of a gel during repeated deformation. The highest cohesiveness observed for CES-DH indicated a stable internal network, probably resulting from strong molecular entanglements and junction zones [57]. Chewiness, calculated from hardness, springiness, and cohesiveness, represented the energy required to chew a gel until it was ready for swallowing. Accordingly, CES-DH also exhibited the highest chewiness, further confirming the formation of a firm, elastic, and coherent gel network. Resilience indicated the ability of a gel to rapidly recover energy after deformation. CES-DH showed the maximum resilience, suggesting a flexible yet stable molecular structure with efficient elastic recovery, which was consistent with its highest springiness and cohesiveness. In summary, the observed differences in gel textural properties among the canna starches from different origins suggested their potential differentiation in starch-based food applications.

### 2.7. In Vitro Digestibility

The in vitro digestibility of the four native, gelatinized, and retrograded canna starches is shown in Table 4. In the native state, all canna starches were dominated by RS, with contents exceeding 80%, indicating a high resistance to enzymatic digestion. Kong et al. [39] reported that native starch granules contained intact crystalline regions, which hindered enzymatic hydrolysis and resulted in a slow digestion rate. Notably, native CES-YX exhibited a significantly higher RS content than the other samples (*p* < 0.05), which might be associated with its higher amylose content. Amylose has been reported to promote interactions with amylopectin, leading to a smoother and more rigid granular surface that restricted the diffusion and adsorption of the enzyme into starch granules [37]. Compared with the native canna starches, the RDS contents of their gelatinized counterparts increased markedly to 70.26%, 78.47%, 81.78%, and 71.60% for CES-YB, CES-MS, CES-DH, and CES-YX, respectively. In contrast, the RS contents decreased substantially and ranged from 0.60% (CES-YX) to 5.56% (CES-MS). Huang et al. [13] indicated that, upon gelatinization, the inter- and intramolecular hydrogen bonds among starch chains were disrupted, causing granule swelling and structural disintegration, thereby increasing the accessibility of starch chains to digestive enzymes. The highest RS content in gelatinized CES-MS might be attributed to its highest lipid content (0.74%), as the formation of amylose–lipid complexes could limit granule swelling during heating and reduce enzyme accessibility [31]. The RS contents of retrograded canna starches were higher than those of gelatinized starches but remained lower than those of native starches. This increase in RS content might be related to partial recrystallization of starch molecules into double-helical structures, resulting in compact molecular assemblies [17]. During the retrogradation of gelatinized starch, the disrupted amylose and amylopectin chains gradually reassociated into partially ordered structures that differed from those of native granules, thereby increasing resistance to enzymatic digestion [58]. In addition, CES-YB exhibited the highest ratio of RS in retrograded starch to that in gelatinized starch, which was consistent with its highest SB value and FV/TV ratio, suggesting a strong tendency toward starch retrogradation.

## 3. Conclusions

This study demonstrated that the different geographical origin of canna starches led to pronounced differences in granule characteristics, molecular structure, crystalline organization, and physicochemical properties. Noticeable variations were observed in granule morphology (oval, round, and disc-shaped shapes), amylose content, chain length distribution, crystalline type (B-type and C-type), and short-range molecular order among the four starches. These structural differences further resulted in distinct thermal behavior, pasting properties, rheological behaviors, and digestion characteristics. These structural–functional relationships provide a basis for the targeted application of canna starches in the food industry. For instance, CES-YB, with its superior final viscosity and high consistency coefficient (*K*), has potential use as a food-thickening and stabilizing agent. In contrast, CES-YX, characterized by its high amylose and resistant starch content, is better suited for formulating functional, low-glycemic-index foods. Looking forward, further studies are needed to better understand how the molecular structure of canna starch influences its functional performance during food processing. In addition, investigating the interactions between canna starch and other food components (such as proteins or lipids) in complex food systems may help expand its potential applications.

## 4. Materials and Methods

### 4.1. Materials

Four canna (*Canna edulis* Ker.) rhizomes were purchased in 2024 from Shiyun E-commerce Co., Ltd. (Liangping District, Chongqing, China). The rhizomes were obtained from plantations in Yibin City and Meishan City (Sichuan Province, China), as well as Dehong Dai and Jingpo Autonomous Prefecture and Yuxi City (Yunnan Province, China). All rhizomes were harvested after approximately 8 months of growth. The isolated starches from these sources were coded as CES-YB, CES-MS, CES-DH, and CES-YX, respectively, where “CES” denoted *Canna edulis* Ker. starch. Specifically, CES-YB and CES-YX represented traditional red-skinned landraces adapted to hilly and plateau climates, respectively; CES-MS was from a white-skinned cultivar “Chuanjiaoyu 3”, and CES-DH was from the cultivar “Qianbeijiaoyu”, grown in the tropical monsoon region.

A series of pullulan standards with molecular weights of 342, 3650, 21,000, 131,400, 610,500, 821,700, and 3,755,000 Da were purchased from Analytical Products & Standards Corporation (Tokyo, Japan). Porcine pancreatic α-amylase (50 U/mg) and amyloglucosidase (3300 U/mL) derived from *Aspergillus niger* were purchased from Sigma-Aldrich (Shanghai, China). Dimethyl sulfoxide (DMSO, ≥99.9%) was obtained from ANPEL Laboratory Technologies (Shanghai, China). Sodium acetate anhydrous (CH_3_COONa, ≥99.5%) was purchased from Energy Chemical Co., Ltd. (Shanghai, China). Sodium azide (NaN_3_, ≥99%) was obtained from Sangon Biotech Co., Ltd. (Shanghai, China). Isoamylase (≥140 U/mg) and lithium bromide (LiBr, ≥99%) were supplied by Shanghai Macklin Biochemical Co., Ltd. (Shanghai, China), and absolute ethanol (C_2_H_6_O, 99.7%) was purchased from Chengdu Kelong Chemical Co., Ltd. (Chengdu, China). A starch assay kit (enzymatic method) and a glucose assay kit (GOPOD method) were purchased from Suzhou Grace Bio-Technology Co., Ltd. (Suzhou, China).

### 4.2. Extraction of Canna Starch

Starch was extracted using the method described by Yuan et al. [17] with some modifications. The canna rhizomes were washed, peeled, and cut into small pieces and then mixed with deionized water at a ratio of 1:4 (*w*/*v*). The mixture was homogenized using a homogenizer (JYL-C16D, Joyoung, Ji’nan, China) to obtain a slurry. The slurry was filtered through an 80-mesh nylon cloth to remove fibrous materials and coarse residues. The filtrate was collected and allowed to stand at 4 °C for 4 h to facilitate starch sedimentation. After decanting the supernatant, the starch precipitate was resuspended in deionized water, and the sedimentation process was repeated five times until the washing water became clear. The purified starch was then collected and dried in an oven (DHG-9240, Shanghai Qixin Scientific Instrument Co., Ltd., Shanghai, China) at 40 °C for 24 h. Finally, the dried starches were ground and passed through a 100-mesh sieve to obtain homogeneous powders. The moisture content of the starches was determined prior to storage, and the samples were then stored in moisture-proof aluminum foil bags for further analysis. The extraction yield of canna starch was not determined in this study.

### 4.3. Measurement of Chemical Composition

The chemical composition of the starch samples was analyzed according to the official methods of AOAC International (22nd edition). Specifically, moisture content was determined by the direct drying method, crude fat content by Soxhlet extraction, crude protein content by the Kjeldahl method, and ash content by incineration at 550 °C. Total starch content was quantified using a starch assay kit. Apparent amylose content (AAC) was determined by the iodine colorimetric method described by Yuan et al. [17].

### 4.4. Color Measurement

The color parameters of the starch samples were measured using a spectrophotometer (CM-5, Konica Minolta, Osaka, Japan) with reference to Yaruro Cáceres et al. [11]. The instrument was calibrated against a standard white plate with values of L* (lightness) = 92.90, a* (redness and greenness) = 0.36, and b* (yellowness and blueness) = −0.08 in order to obtain the L*, a*, and b* values of the starch samples. The whiteness index (*WI*) was calculated according to Equation (1):

(1) $$\mathrm{WI}=100-\sqrt{{(100-L^{*})}^{2}+{\left(a^{*}\right)}^{2}+{\left(b^{*}\right)}^{2}}$$

### 4.5. Scanning Electron Microscopy (SEM)

A small amount of starch sample was mounted on a sample stage using double-sided adhesive tape and subsequently gold-coated by sputtering for 60 s under vacuum conditions. The morphology of the starch granules was examined using a field emission scanning electron microscope (Sigma 360, Zeiss Gemini, Oberkochen, Germany) at an accelerating voltage of 3 kV. Microstructural images of the starch granules were captured at magnifications of 300× and 500×. The overall size of starch granules was calculated using the ImageJ software (version 1.54, National Institutes of Health, Bethesda, MD, USA).

### 4.6. Determination of Particle Size Distribution

According to the protocol of Tao et al. [26], the particle size distribution of the starch samples was measured using a laser diffraction particle size analyzer (Mastersizer 3000, Malvern, UK). At the beginning of the measurement, starch samples were dispersed in a beaker containing approximately 500 mL of deionized water and stirred at a speed of 2000 r/min to ensure that the coverage of all samples was maintained within the range of 8% to 20%. The particle size was characterized by *D*_10_, *D*_50_, and *D*_90_, which represented the particle diameters corresponding to 10%, 50%, and 90% of the cumulative volume, respectively. *D*_[4,3]_ and *D*_[3,2]_ represented the volume-weighted mean diameter and surface-area-weighted mean diameter, respectively. The *Span* of the particle size distribution, indicating the degree of dispersion, was calculated according to Equation (2) described by Sánchez et al. [27]:

(2) $$\mathrm{Span}=\frac{D_{90}-D_{10}}{D_{50}}$$

### 4.7. X-Ray Diffraction (XRD)

The starch samples were placed in a dryer to balance moisture at room temperature for 24 h before testing. The crystalline structure of the samples was determined by using an X-ray diffractometer (X’Pert3 Powder, PANalytical B.V., The Netherlands) following the method of Tao et al. [30] with minor modifications. The measurement conditions were as follows: Cu–Kα radiation source (*λ* = 0.154 nm), voltage of 40 kV, current of 40 mA, scanning speed of 4°/min, step size of 0.02°, and a diffraction angle (2*θ*) range of 4–40°. The relative crystallinity (RC, %) of the samples was calculated according to Equation (3) by using the MDI Jade 6.5 software (Materials Data Inc., Livermore, CA, USA), referring to the calculation method of Tao et al. [30].

(3) $$\mathrm{RC}\;\left(\%\right)=\frac{A_{c}}{A_{c}+A_{a}}\times 100$$

where *A*_c_ and *A*_a_ represent the peak area of the crystalline zone and area of the amorphous zone, respectively.

### 4.8. Fourier Transform Infrared Spectroscopy (FTIR)

Based on the method reported by Yan et al. [5], the starch samples were evenly spread on the surface of the attenuated total reflectance (ATR) accessory (Spectrum100, PerkinElmer, Shelton, CT, USA) equipped with a diamond crystal for analysis. The measurement parameters were set as follows: a resolution of 4 cm^−1^, 32 scan accumulations, and a spectral range of 4000–600 cm^−1^. The resulting spectra were deconvoluted using the OMNIC 8.0 software (Thermo Nicolet Corp., Erie, PA, USA), and the peak intensities at 1047 cm^−1^, 1022 cm^−1^, and 995 cm^−1^ were obtained. Subsequently, the intensity ratios *R*_1047/1022_ and *R*_995/1022_ were calculated to assess the short-range ordered structure of the starch samples.

### 4.9. Determination of Chain Length Distribution

Debranching of canna starch was performed following the method of Xing et al. [19] with minor modifications. A 5 mg starch sample was suspended in 0.9 mL of deionized water and gelatinized in a boiling water bath for 15 min with intermittent vortexing. After cooling to room temperature, 0.1 mL of CH_3_COONa buffer (0.1 mol/L, pH = 3.5), 5 μL of NaN_3_ solution (40 mg/mL), and 10 μL of isoamylase were added. The mixture was incubated in a water bath at 37 °C for 3 h. The reaction was terminated by adding 5 mL of absolute ethanol, followed by centrifugation at 4000× *g* for 10 min. The precipitate was dissolved in 1 mL of DMSO/LiBr solution at 80 °C with shaking (350 r/min) for 2 h.

The chain length distribution of the debranched starch was analyzed using a size-exclusion chromatography (SEC) system (Ultimate3000, Thermo Fisher Scientific, Waltham, MA, USA USA) equipped with a refractive index detector (Optilab T-rEX, Wyatt Technology Co., Santa Barbara, CA, USA). Separation was performed on two columns (MIXED-B, 300 × 7.5 mm, 10 μm; and MIXED-D, 300 × 7.5 mm, 5 μm; Agilent Technologies Inc., Santa Clara, CA, USA) maintained at 80 °C. The injection volume was 100 μL, and the mobile phase consisted of 0.5% LiBr in DMSO at a flow rate of 0.5 mL/min. The columns were calibrated with pullulan standards. In the SEC distribution curve, amylose long chains and amylopectin short chains were clearly separated at a degree of polymerization (DP) of approximately 100. The true amylose content (TAC) was calculated as the ratio of the area under the curve for DP > 100 to the total area of the curve.

### 4.10. Determination of Solubility and Swelling Power

According to the method mentioned by Kong et al. [53] with minor modifications, a starch sample (0.2000 g, on a dry basis, denoted as *m*_0_) was accurately weighed into a 50 mL centrifuge tube. Deionized water was added to formulate a starch dispersion with a concentration of 2% (*w*/*w*). The suspension was heated with magnetic stirring for 30 min in a water bath at temperatures ranging from 55 °C to 95 °C in 10 °C increments. After cooling to room temperature, the samples were centrifuged at 6790× *g* for 30 min using a centrifuge (H1850R, Xiangyi Centrifuge Instrument Co., Ltd., Changsha, China). The supernatant was carefully decanted into a pre-weighed drying dish and dried to a constant weight (*m*_1_, g) at 105 °C in a drying oven (DHG-9240, Shanghai Qixin Scientific Instrument Co., Ltd., Shanghai, China). The mass of the precipitate remaining in the centrifuge tube was recorded as *m*_2_ (g). The solubility (*SOL*, %) and swelling power (*SP*, g/g) were calculated according to Equations (4) and (5), respectively.

(4) $$\mathrm{SOL}\;(\%)=\frac{m_{1}}{m_{0}}\times 100$$

(5) $$\mathrm{SP}\;(g/g)=\frac{m_{2}}{m_{0}\times (1-\mathrm{SOL})}$$

### 4.11. Differential Scanning Calorimetry (DSC)

The thermal properties of the canna starches were examined by a differential scanning calorimeter (DSC 4000, PerkinElmer, Waltham, MA, USA). A starch sample of 0.0030 g was accurately weighed and mixed with deionized water at a 1:2 (*w*/*w*) ratio in an aluminum pan. The mixture was sealed and equilibrated at room temperature for 24 h to allow for full hydration of the starch. The sample was then heated from 10 °C to 120 °C at a rate of 10 °C/min. The onset temperature (*T*_o_), peak temperature (*T*_p_), conclusion temperature (*T*_c_), gelatinization temperature range (Δ*T* = *T*_c_ − *T*_o_) and enthalpy of gelatinization (Δ*H*, were obtained from the DSC thermogram.

### 4.12. Rapid Viscosity Analyzer (RVA)

A starch–water slurry (5.36%, *w*/*w*) was prepared by accurately weighing 1.5 g of starch sample (dry basis) and 26.5 g of deionized water into an aluminum canister. The pasting properties of the samples were determined using a Rapid Visco Analyzer (RVA-TecMaster, Perten Scientific Instruments Ltd., Stockholm, Sweden). The test parameters were as follows: The paddle rotating speed was set to 960 r/min during the initial 10 s and then reduced to 160 r/min for the remainder of the measurement. The slurry was equilibrated at 50 °C for 1 min, heated to 95 °C at a rate of 10 °C/min, held at 95 °C for 2.5 min, and then cooled to 50 °C at a rate of 12 °C/min. Finally, the sample was held at 50 °C for 2 min. Pasting parameters, including peak viscosity (PV), trough viscosity (TV), breakdown viscosity (BD), final viscosity (FV), and setback viscosity (SB), were obtained using TCW 3, the proprietary software provided with the instrument.

### 4.13. Determination of Rheological Properties

A rotational rheometer (DRH-2, TA Instruments, New Castle, DE, USA) equipped with a parallel plate geometry (40 mm in diameter), was used to determine the rheological properties of the canna starch samples. The rheometer parallel plate was preheated to 50 °C, and the gap to the parallel plate was set to 1 mm. The starch–water slurry (5.36%, *w*/*w*) was heated in a 95 °C water bath with continuous stirring for 30 min to obtain a starch paste, and then the hot starch paste was immediately transferred to a parallel plate. The upper plate was lowered to the preset gap, excess paste around the edges was carefully trimmed, and a thin layer of silicone oil was applied around the sample to prevent water evaporation. After loading, the sample was allowed to equilibrate for 60 s to eliminate residual stress and stabilize the temperature (50 °C) before the rheological test was initiated. The equilibration time was determined based on preliminary tests.

#### 4.13.1. Steady Flow Measurement of Starch Pastes

With the increase in shear rate (0.01 to 100 s^−1^), the apparent viscosity (*η*_ap_) and shear stress (*τ*) were recorded as a function of the shear rate (*γ*). The experimental data were fitted using the Herschel–Bulkley model, as expressed in Equation (6):

(6) $$\tau =\tau_{0}+K\gamma^{n}$$

where *τ* is the shear stress (Pa), *τ*_0_ is the yield stress (Pa), *γ* is the shear rate (s^−1^), *K* is the consistency coefficient (Pa·s*^n^*), and *n* is the flow behavior index.

#### 4.13.2. Dynamic Frequency Sweep Measurement of Starch Pastes

The amplitude sweep was first conducted over a strain range of 0.01% to 100% at a constant frequency of 1 Hz to determine the linear viscoelastic region (LVR) of the starch paste. Frequency sweep measurements were then performed within the LVR using a strain of 2% and an angular frequency (*ω*) ranging from 0.1 to 100 rad/s. The storage modulus (*G*′), loss modulus (*G*″), and loss tangent (tan *δ* = *G*″/*G*′) were recorded as a function of *ω*. The variations in *G*′ and *G*″ with *ω* were fitted using power-law model equations according to the method of Kong et al. [53]:

(7) $$G^{\prime }\left(\omega \right)=K^{\prime }\cdot \omega^{a}$$

(8) $$G^{\prime \prime }\left(\omega \right)=K^{\prime \prime }\cdot \omega^{b}$$

where *ω* is the angular frequency (rad/s), *K*′ and *K*″ represent the respective moduli at an angular frequency of 1 rad/s, while the exponents *a* and *b* quantify the frequency dependence of *G*′(*ω*) and *G*″(*ω*), respectively.

### 4.14. Determination of Texture Properties of Starch Gels

The textural properties of the canna starch gels were assessed using a texture analyzer (TA. XT plus, Stable Micro Systems, Godalming, UK). The starch–water slurry (5.36%, *w*/*w*) was prepared to match the concentration used in the RVA and rheological measurements and was heated in a 95 °C water bath with continuous stirring for 30 min to obtain a starch paste, and the paste was then poured into silicone molds (3 cm in diameter and 1.8 cm in height) and stored at 4 °C for 24 h to allow for gel formation. Texture profile analysis was performed using a cylindrical probe (P36/R). The pre-test, test, and post-test speeds were all set at 1 mm/s. The trigger force was 5 g, the compression ratio was 50%, and the time interval between the two compressions was 5 s. The determination of each sample was independently repeated 8 times. Textural parameters, including hardness, springiness, adhesiveness, cohesiveness, chewiness, and resilience were obtained using the Exponent software (version 6.2.7.0, Stable Micro Systems, Godalming, UK) equipped with the instrument.

### 4.15. In Vitro Digestion Analysis of Starch

The *in vitro* digestibility of the native, gelatinized, and retrograded starch was assessed based on the methods described by Yuan et al. [17] with some modifications. Rapidly digestible starch (RDS), slowly digestible starch (SDS), and resistant starch (RS) contents were determined. Briefly, a 200 mg starch sample was suspended in 10 mL of CH_3_COONa buffer (0.2 mol/L, pH = 5.2), and gelatinization was achieved by heating the suspension in boiling water for 30 min. To prepare retrograded starch, the gelatinized sample was stored at 4 °C for 48 h. A total of 0.5 g of porcine pancreatic α-amylase (50 U/mg) was suspended in 10 mL of CH_3_COONa buffer (0.2 mol/L, pH = 5.2) with magnetic stirring for 10 min and then centrifuged at 1500× *g* for 5 min. The supernatant was transferred into a beaker and mixed with 0.4 mL amyloglucosidase (3300 U/mL) before use. The starch samples (2%, *w*/*v*) were incubated in a shaking water bath at 37 °C and 200 r/min for 10 min prior to the addition of 0.5 mL of the enzyme mixture. Enzymatic hydrolysis was then carried out under the same conditions. At 0, 20, and 120 min, 0.5 mL of the reaction mixture was collected and immediately mixed with 2 mL of absolute ethanol to terminate the reaction. The mixture was then centrifuged at 4000× *g* for 10 min, and the glucose concentration in the supernatant was determined using a GOPOD assay kit. RDS, SDS, and RS contents were calculated using Equations (9), (10), and (11), respectively:

(9) $$\mathrm{RDS}\;(\%)=\frac{\left(G_{20}-\mathrm{FG}\right)\times 0.9}{\mathrm{TS}}\times 100$$

(10) $$\mathrm{SDS}\;\left(\%\right)=\frac{\left(G_{120}-G_{20}\right)\times 0.9}{\mathrm{TS}}\times 100$$

(11) RS (%)=100%−RDS−SDS

where *G*_20_ and *G*_120_ represent the amounts of glucose (mg) released after 20 and 120 min of enzymatic hydrolysis, respectively; *FG* is the amount of free glucose in the sample before enzymatic hydrolysis (mg); and *TS* is the total starch content in the sample (mg).

### 4.16. Statistical Analysis

The measurements were conducted in triplicate (unless otherwise stated), and the results were presented as means ± standard deviations. One-way analysis of variance and Duncan’s multiple range test were performed using the SPSS 27.0 software (IBM Corp., Armonk, NY, USA), and significant differences were considered at *p* < 0.05. Graphs and Pearson’s correlation analysis were generated using Origin 2025 (OriginLab Corporation, Northampton, MA, USA).

## Figures and Tables

**Table 1:** Proximate composition, color values, and particle size distribution of canna starches from different geographical regions in China.

Parameters	CES-YB	CES-MS	CES-DH	CES-YX
Proximate composition				
Moisture (%)	16.02 ± 0.04 ^b^	18.19 ± 0.03 ^a^	15.37 ± 0.08 ^c^	11.87 ± 0.03 ^d^
Total starch (%)	86.67 ± 0.47 ^c^	89.83 ± 0.56 ^b^	82.51 ± 0.68 ^d^	93.22 ± 1.86 ^a^
Apparent amylose content (%)	33.93 ± 0.35 ^c^	31.44 ± 0.13 ^d^	36.61 ± 0.55 ^b^	43.62 ± 0.76 ^a^
True amylose content (%)	29.23 ± 0.16 ^b^	23.38 ± 0.22 ^c^	15.21 ± 0.22 ^d^	35.90 ± 0.11 ^a^
Lipid (%)	0.50 ± 0.00 ^b^	0.74 ± 0.02 ^a^	0.44 ± 0.00 ^c^	0.22 ± 0.02 ^d^
Protein (%)	0.16 ± 0.01 ^b^	0.10 ± 0.02 ^bc^	0.06 ± 0.03 ^c^	0.38 ± 0.05 ^a^
Ash (%)	0.26 ± 0.01 ^b^	0.46 ± 0.01 ^a^	0.18 ± 0.03 ^c^	0.11 ± 0.02 ^d^
Color value				
L*	92.93 ± 0.03 ^c^	94.82 ± 0.02 ^b^	92.63 ± 0.03 ^d^	97.79 ± 0.04 ^a^
a*	0.73 ± 0.03 ^a^	−0.10 ± 0.01 ^c^	0.08 ± 0.02 ^b^	−0.36 ± 0.02 ^d^
b*	5.68 ± 0.04 ^a^	1.21 ± 0.02 ^c^	4.20 ± 0.02 ^b^	1.10 ± 0.03 ^d^
*WI*	90.90 ± 0.04 ^d^	94.68 ± 0.02 ^b^	91.52 ± 0.02 ^c^	97.51 ± 0.03 ^a^
Particle size distribution				
*D*_10_ (μm)	11.25 ± 0.41 ^d^	26.60 ± 0.42 ^b^	29.73 ± 0.45 ^a^	12.53 ± 0.07 ^c^
*D*_50_ (μm)	39.23 ± 0.76 ^c^	43.71 ± 1.12 ^b^	48.35 ± 0.59 ^a^	20.19 ± 0.03 ^d^
*D*_90_ (μm)	65.70 ± 0.73 ^b^	67.94 ± 2.74 ^b^	74.57 ± 0.86 ^a^	31.33 ± 0.18 ^c^
*Span*	1.39 ± 0.04 ^a^	0.94 ± 0.04 ^b^	0.93 ± 0.01 ^b^	0.93 ± 0.01 ^b^
*D*_[4,3]_ (μm)	39.52 ± 0.52 ^c^	45.65 ± 1.33 ^b^	50.42 ± 0.64 ^a^	21.16 ± 0.05 ^d^
*D*_[3,2]_ (μm)	22.97 ± 0.50 ^c^	40.37 ± 0.87 ^b^	44.83 ± 0.56 ^a^	18.81 ± 0.04 ^d^

Values are presented as mean ± standard deviation (*n* = 3). Values in the same row with different lowercase letters indicate significant differences (*p* < 0.05). Moisture content was determined on a wet basis, whereas other components are expressed on a dry weight basis. The apparent amylose content was calculated by the iodine colorimetric method; the true amylose content is the ratio of the area under the curve for DP > 100 to the total area of the chain length distribution curve; L*, lightness; a*, redness (+) and greenness (−); b*, yellowness (+) and blueness (−); *WI*, whiteness index; *D*_10_, *D*_50_, and *D*_90_ represent the particle diameters at 10%, 50%, and 90% of the cumulative volume, respectively; *Span* indicates the dispersion of the particle size distribution; *D*_[4,3]_ and *D*_[3,2]_ denote the volume-weighted mean diameter and surface area-weighted mean diameter, respectively.

**Table 2:** Structural parameters of canna starches from four different geographical regions in China.

Parameters	CES-YB	CES-MS	CES-DH	CES-YX
Long-range ordered structure				
RC (%)	23.70 ± 0.18 ^b^	25.36 ± 0.21 ^a^	21.55 ± 0.19 ^c^	20.53 ± 0.40 ^d^
Short-range ordered structure				
*R*_1047/1022_	0.59 ± 0.00 ^c^	0.63 ± 0.00 ^a^	0.61 ± 0.01 ^b^	0.56 ± 0.01 ^d^
*R*_995/1022_	1.21 ± 0.01 ^c^	1.26 ± 0.00 ^a^	1.23 ± 0.01 ^b^	1.15 ± 0.01 ^d^
GPC peak area				
Peak 1 (%)	51.74	53.36	59.67	52.30
Peak 2 (%)	25.85	32.78	40.33	16.10
Peak 3 (%)	22.41	13.86	0.00	31.60
The branching degree of amylopectin	2.00	1.63	3.25	1.48

Values are presented as mean ± standard deviation (*n* = 3). Values in the same row with different lowercase letters are significantly different (*p* < 0.05). RC, relative crystallinity; *R*_1047/1022_, the ratio of absorbance at 1047 cm^−1^ to 1022 cm^−1^; *R*_995/1022_, the ratio of absorbance at 995 cm^−1^ to 1022 cm^−1^; Peak 1 and Peak 2 correspond to the short chains (A-chains and short B-chains) and medium-to-long chains (long B-chains) of amylopectin, respectively; Peak 3 represents amylose; the area ratio of Peak 1 to Peak 2 reflects the branching degree of amylopectin.

**Table 3:** Thermal, pasting, rheological and texture properties of canna starches from four different geographical regions in China.

Parameters	CES-YB	CES-MS	CES-DH	CES-YX
Thermal properties				
*T*_o_ (°C)	61.30 ± 0.28 ^b^	63.13 ± 0.25 ^a^	62.33 ± 0.00 ^ab^	61.82 ± 0.78 ^b^
*T*_p_ (°C)	66.70 ± 0.02 ^a^	67.28 ± 0.36 ^a^	67.61 ± 0.56 ^a^	66.68 ± 0.15 ^a^
*T*_c_ (°C)	77.36 ± 0.02 ^a^	73.63 ± 0.08 ^b^	74.76 ± 1.52 ^b^	70.96 ± 0.44 ^c^
∆*T* (°C)	16.06 ± 0.30 ^a^	10.5 ± 0.17 ^bc^	12.43 ± 1.52 ^b^	9.14 ± 0.35 ^c^
∆*H*_g_ (J/g)	3.53 ± 0.01 ^a^	2.92 ± 0.20 ^b^	2.82 ± 0.01 ^b^	0.43 ± 0.09 ^c^
Pasting properties				
PV (cP)	1572.50 ± 82.73 ^c^	2086.50 ± 60.10 ^b^	2721.50 ± 70.00 ^a^	399.50 ± 7.78 ^d^
TV (cP)	1433.50 ± 60.10 ^c^	1847.00 ± 11.31 ^a^	1633.00 ± 4.24 ^b^	376.00 ± 11.31 ^d^
BD (cP)	139.00 ± 22.63 ^bc^	239.50 ± 71.42 ^b^	1088.50 ± 65.76 ^a^	23.50 ± 3.54 ^c^
FV (cP)	2147.50 ± 27.58 ^b^	2413.50 ± 26.12 ^a^	2091.00 ± 16.97 ^b^	518.50 ± 13.44 ^c^
SB (cP)	714.00 ± 32.53 ^a^	566.50 ± 37.47 ^b^	458.00 ± 12.73 ^c^	142.50 ± 2.12 ^d^
Parameters from steady flow measurement				
*τ*_0_ (Pa)	11.38 ± 0.85 ^a^	7.43 ± 0.88 ^b^	7.28 ± 1.07 ^b^	2.84 ± 0.15 ^c^
*K* (Pa·s^n^)	24.42 ± 0.22 ^a^	17.78 ± 2.15 ^b^	13.38 ± 1.01 ^c^	5.08 ± 0.04 ^d^
*n*	0.38 ± 0.01 ^c^	0.49 ± 0.01 ^ab^	0.52 ± 0.01 ^a^	0.48 ± 0.01 ^b^
*R* ^2^	0.984	1.000	0.996	1.000
Parameters from dynamic frequency sweep measurement				
*K*′ (Pa)	94.08 ± 11.98 ^a^	32.80 ± 4.92 ^b^	27.87 ± 2.38 ^bc^	11.88 ± 2.14 ^c^
*a*	0.08 ± 0.02 ^d^	0.26 ± 0.01 ^b^	0.19 ± 0.00 ^c^	0.34 ± 0.04 ^a^
*R* ^2^	0.975	0.984	0.996	0.979
*K*″ (Pa)	9.00 ± 2.43 ^ab^	10.38 ± 0.78 ^a^	8.01 ± 0.23 ^ab^	6.01 ± 0.56 ^b^
*b*	0.40 ± 0.03 ^a^	0.28 ± 0.00 ^b^	0.30 ± 0.01 ^b^	0.32 ± 0.01 ^b^
*R* ^2^	0.988	0.998	0.989	0.999
Texture properties				
Hardness (g)	2555.00 ± 10.82 ^c^	1461.17 ± 10.29 ^d^	3250.83 ± 32.85 ^a^	2761.06 ± 68.45 ^b^
Springiness	0.92 ± 0.02 ^b^	0.91 ± 0.00 ^b^	0.96 ± 0.01 ^a^	0.86 ± 0.00 ^c^
Adhesiveness (g·s)	−217.79 ± 16.81 ^a^	−375.31 ± 12.10 ^b^	−852.14 ± 43.22 ^d^	−420.92 ± 18.66 ^c^
Cohesiveness	0.83 ± 0.01 ^b^	0.79 ± 0.00 ^c^	0.96 ± 0.01 ^a^	0.33 ± 0.00 ^d^
Chewiness (mJ)	1891.03 ± 41.86 ^b^	968.68 ± 12.22 ^d^	2866.61 ± 70.19 ^a^	1157.59 ± 13.81 ^c^
Resilience	0.25 ± 0.00 ^c^	0.28 ± 0.01 ^b^	0.31 ± 0.00 ^a^	0.07 ± 0.00 ^d^

Values are presented as mean ± standard deviation (*n* = 3). Values within the same row with different lowercase letters are significantly different (*p* < 0.05). *T*_o_, *T*_p_, and *T*_c_ represent onset, peak, and conclusion gelatinization temperatures, respectively; ∆*T*, the difference between *T*_o_ and *T*_c_; ∆*H*_g_, enthalpy of gelatinization; PV, TV, BD, FV and SB mean pasting viscosity, trough viscosity, breakdown viscosity, final viscosity, and setback viscosity, respectively; *τ*_0_, *K*, *n*, and *R*^2^ indicate the yield stress, consistency coefficient, flow behavior index and coefficient of determination for measuring the fitting effect, respectively; *K*′, *K*″, *a* and *b* represent the storage modulus, loss modulus, and dimensionless exponents that quantify their dependences on oscillation frequency, respectively.

**Table 4:** In vitro digestion properties of native, gelatinized and retrograded canna starches from four different geographical regions in China.

Parameters	CES-YB	CES-MS	CES-DH	CES-YX
Native starch				
RDS (%)	10.30 ± 0.13 ^b^	12.10 ± 0.38 ^a^	9.60 ± 0.39 ^b^	8.26 ± 0.73 ^c^
SDS (%)	7.08 ± 0.10 ^a^	4.95 ± 0.50 ^bc^	5.09 ± 0.27 ^b^	3.46 ± 0.95 ^c^
RS (%)	82.61 ± 0.03 ^b^	82.95 ± 0.89 ^b^	85.31 ± 0.66 ^b^	88.29 ± 1.68 ^a^
Gelatinized starch				
RDS (%)	70.26 ± 0.17 ^b^	78.47 ± 1.80 ^a^	81.78 ± 2.58 ^a^	71.60 ± 0.64 ^b^
SDS (%)	29.13 ± 0.36 ^a^	15.97 ± 1.68 ^b^	16.91 ± 2.34 ^b^	26.50 ± 1.86 ^a^
RS (%)	0.60 ± 0.19 ^b^	5.56 ± 0.12 ^a^	1.31 ± 0.25 ^b^	1.89 ± 1.21 ^b^
Retrograded starch				
RDS (%)	69.12 ± 0.63 ^a^	64.06 ± 0.22 ^b^	70.45 ± 0.68 ^a^	62.68 ± 0.02 ^c^
SDS (%)	16.18 ± 0.76 ^c^	19.80 ± 0.42 ^b^	20.62 ± 0.49 ^ab^	21.64 ± 0.66 ^a^
RS (%)	14.69 ± 0.14 ^b^	16.14 ± 0.20 ^a^	8.92 ± 0.19 ^c^	15.68 ± 0.68 ^ab^

Values are presented as mean ± standard deviation (*n* = 3). Values within the same row with different lowercase letters are significantly different (*p* < 0.05). RDS, rapidly digestible starch; SDS, slowly digestible starch; RS, resistant starch.

## Data Availability

Data will be made available on request.
